# *In vitro* investigation of the impact of remaining tooth structure on the tensile failure loads of overdenture copings

**DOI:** 10.4317/jced.56228

**Published:** 2019-11-01

**Authors:** Anna Fotiou, Savvas N. Kamalakidis, Argirios L. Pissiotis, Konstantinos Michalakis

**Affiliations:** 1 DDS. Resident, Department of Prosthodontics, Aristotle University Faculty of Health Sciences, School of Dentistry, Thessaloniki, Greece; 2 DDS, PhD, FACP. Faculty, Department of Prosthodontics, Aristotle University Faculty of Health Sciences, School of Dentistry, Thessaloniki, Greece and Adjunct Assistant Professor, Division of Postgraduate Prosthodontics, Tufts University School of Dental Medicine, Boston, Mass; 3 DDS, MS, PhD. Professor, Department of Prosthodontics, Aristotle University Faculty of Health Sciences, School of Dentistry, Thessaloniki, Greece; 4 DDS, MSc, PhD, FACP. Associate Professor and Director of Graduate Prosthodontics, Aristotle University Faculty of Health Sciences, School of Dentistry, Thessaloniki, Greece and Adjunct Associate Professor, Division of Postgraduate Prosthodontics, Tufts University School of Dental Medicine, Boston, Mass

## Abstract

**Background:**

The purpose of this *in vitro* study was to evaluate the impact of the remaining tooth structure on the retention of overdenture cast metal copings.

**Material and Methods:**

A freshly extracted intact mandibular human canine (length 25 mm) was selected and endodontically treated. An incisal reduction of 4 mm with no ferrule preparation was performed and a post space of 12 mm was created. By using polyvinyl siloxane duplication material and autopolymerizing polymethylmethacrylate resin, ten resin teeth analogs (control group) were obtained. A second set of ten resin teeth analogs (group 1) was created by preparing on the original natural tooth a 360o ferrule design of 1 mm in height and by using the same procedural technique. The canine was further reduced by an additional 1 mm, resulting in a ferrule of 2 mm in height, measured from the initial incisal reduction, thus a third set of ten resin teeth analogs (group 2) was created. For every tooth analog in all groups a dome-shaped metal coping was cast and luted with a glass ionomer cement. All specimens were subjected to tensile load testing until decementation occurred.

**Results:**

The specimens in the control group exhibited a mean failure load of 87.21 ± 18.26 N, while the 1 mm ferrule group recorded a higher mean failure load of 125.43 ± 8.79 N and the 2 mm ferrule group recorded the highest mean failure load of 146.12 ± 23.38 N. One-way ANOVA revealed significant differences (F= 28.04, *p*<0.001) in the tensile failure loads between all of the groups being tested.

**Conclusions:**

The metal copings with a 2 mm ferrule design exhibited the highest retention values, followed by the 1 mm ferrule and the no ferrule design, with the differences among them being statistically significant.

** Key words:**Ferrule effect, In vitro study, Metal copings, Overdenture, Tensile stress.

## Introduction

Tooth-supported overdentures have been advocated as a viable alternative to conventional complete denture therapy, since Miller introduced the clinical concept of retaining the patients’ remaining teeth under a removable prosthesis (1). Later, in 1969, Morrow *et al.* (2,3) and Lord and Teel (4) described the simplified procedures needed for the construction of tooth-supported overdentures, thus paving the way for the decade of overdentures in the 1970s (5).

It has been documented that the main advantage of overdenture treatment is the delay of residual ridge resorption by preserving strategic teeth roots after elective devitalization (6,7). Additionally, the preservation of remaining teeth’s periodontal receptors could enhance the patients’ neuromuscular coordination and tactile sensation, adding to the stability of the overdenture prosthesis (8). It has also been postulated that the increased stability may also provide improved masticatory ability and chewing efficiency (9). Furthermore, the psychological benefits of the postponement of natural teeth’s extraction could add to the patients’ higher satisfaction levels (10).

Nevertheless, disadvantages of the overdenture treatment have also been reported in the dental literature. These include periodontal disease and caries (11). Both biological complications have been well documented in numerous studies (12-19). The use of topical antimicrobial agents, regular recall appointments and meticulous daily oral hygiene have proven to be beneficial for the long term prognosis of the underlying teeth (20). Prosthetic complications in relation to the overdenture prostheses and the underlying copings have been thoroughly described (21). These technical complications could vary from denture base fracture or perforation of the denture over the abutment teeth to metal coping refabrication or coping decementation (22).

In order to protect the overdenture abutments, extracoronal restorations, constructed by either composite resin or metal restorative materials, have been advocated (23). This protective coverage was shown to be beneficial for caries protection, since the dentin close to the pulp has been proven to be less calcified and more porous than dentin near the tooth surface (24). When additional retention for the removable prosthesis is required, the incorporation of precision attachments on to the metal copings should be considered (25). The shape design of the overdenture copings could either be the “classical” flat design, dome-shaped, or conical, with an equivalent remaining dentin height and shape of the abutment teeth (26). That remaining tooth structure -ferrule effect- for endodontically treated teeth has been thoroughly described and extensively researched in the dental literature (27,28). It has been shown to protect the integrity of the teeth under complete coverage restorations and promote the longevity of the restorations (29).

Since overdenture abutment copings could be considered as complete coverage restorations, we should expect that incorporating a ferrule design preparation would also be beneficial for the patients’ prosthetic rehabilitation scheme. The purpose of the present *in vitro* study was to evaluate the presence and the height of the ferrule design on the retention values of overdenture cast metal copings. The null hypothesis was that no difference is anticipated in retention loads between the copings with and without a ferrule design.

## Material and Methods

A freshly extracted intact mandibular human canine was selected for this *in vitro* study. The tooth was examined under a stereomicroscope to ensure the absence of cracks and microfractures and was also radiographed to exclude the presence of carious lesions and internal root resorption. Measured labially at the cementoenamel junction (CEJ) the length of the crown was 9 mm and that of the root 16 mm. Following disinfection with a 5.25% hypochlorite solution for 1 hour, the tooth was stored in a 0.9% NaCl isotonic saline solution. The study was conducted in accordance with the Declaration of Helsinki and was approved by the Institutional Review Board/Ethics Committee of the Aristotle University of Thessaloniki School of Dentistry (Protocol Number: 8/03-07-2019). All experimental procedures were performed at the Department of Basic Dental Sciences, Division of Dental Tissue Pathology and Therapeutics, School of Dentistry, Faculty of Health Sciences, Aristotle University of Thessaloniki, Greece.

Access cavity preparation was performed using a round diamond bur (#801-021C; SS White, Lakewood, NJ) and apical patency was verified with a size 10 K-file (K-files; Dentsply-Maillefer, Ballaigues, Switzerland). The working length of the tooth was determined visually by subtracting 1 mm from the length of an ISO size 10 K-file placed at the apical foramen. Endodontic instrumentation of the root canal was performed by using rotary Ni-Ti ProTaper files Sx-F4 (Pro Taper; Dentsply-Maillefer, Ballaigues, Switzerland). The root canal was obturated using lateral compaction of gutta-percha cones (Roeko; Coltene/Whaledent AG, Altstaetten, Switzerland) and an endodontic sealer (AH-26; Dentsply-Maillefer, Ballaigues, Switzerland). The access cavity was sealed with a glass ionomer restorative material (Ketac-Molar; 3M/ESPE, St. Paul, MN) and the tooth was stored in 0.1% thymol solution for five days.

Following that period, the canine was initially reduced incisally by 5 mm, resulting in a 4 mm crown height measured labially from the CEJ with no ferrule preparation. Tooth reduction was performed using medium and fine grit parallel chamfer diamond burs (881.FG.010; Komet USA LLC, Rock Hill, SC), measuring 1.0 mm in diameter. A post space of 12 mm in length was subsequently prepared with Gates-Glidden drills #1-4 (Gates-Glidden; Henry Schein, Inc., New York, NY), obtaining an apical gutta percha seal length of 7 mm. The canine specimen was then positioned vertically by means of a surveyor (Ney Surveyor; Dentsply Inc., York, PA) at the center of a cylindrical plastic mold (30 mm in diameter and in height). The plastic mold was poured with laboratory polyvinyl siloxane (Deguform; Degudent GmbH, Hanau-Wolfgang, Germany) material to obtain the impression of the prepared tooth. Autopolymerizing polymethylmethacrylate (PMMA) resin (Pattern Resin LS; GC America, Alsip, IL) was proportioned and mixed according to the manufacturer’s instructions. It was then poured under vibration (Vibrator 200; Buffalo Dental Mfg Co, Syosset, NY) into the impression and placed in a pressure chamber (Wiropress SL; Bego, Bremen, Germany) under 300 kPa for 20 minutes (30).

Ten acrylic resin patterns (control group) were fabricated with modulus of elasticity similar to that of dentin. Each one of them was placed vertically at the center of a cylindrical plastic mold. The molds were then poured with autopolymerizing polymethylmethacrylate acrylic resin (Vertex; Vertex-Dental BV, Soesterberg, Netherlands) 2 mm below the CEJ of the resin teeth. The metal copings for the study were fabricated directly on the resin teeth using plastic burnout posts (Directa AB, Upplands Vasby, Sweden) and modelling and cervical wax (Thowax; Yeti Dental, Engen, Germany). The wax patterns were dome-shaped with a wax loop incorporated at the coronal part of the pattern to assist in the tensile stress test. They were then invested with phosphate bonded investment (Fujivest II; GC America, Alsip, IL) and cast with a nickel chromium alloy (Wiron Light; Bego, Bremen, Germany). Following the necessary laboratory procedures (i.e. casting, devesting, cleaning), the metal copings were air-abraded with 50-μm aluminum oxide particles under 2.8 kg/cm2 pressure and steam cleaned.

**Figure 1:**
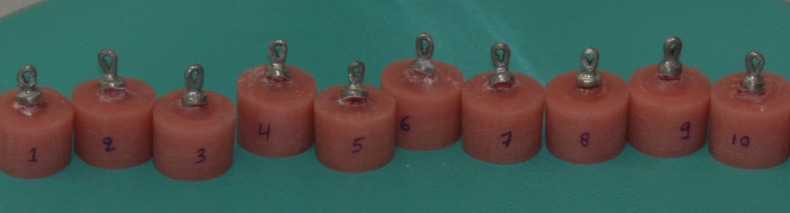
Resin-teeth specimens with cemented metal copings.

For the fabrication of the second group of specimens (n=10) the same mandibular canine was further prepared with a 360o ferrule design 1 mm in height, measured from the previous incisal reduction. The diameter of the diamond was used to control the preparation depth of 1.0 mm. The procedures and materials for the fabrication of the second group were identical with these of the control group. Following the completion for the second group of metal copings, the same canine tooth was further reduced by an additional 1 mm, resulting in a ferrule of 2 mm in height, measured from the initial incisal reduction. The resulting third group of specimens (n=10) was completed with the fabrication of the final metal copings (Fig. 1).

The metal copings of all three groups were cemented to their respective resin tooth analogs with a glass ionomer cement (Ketac Cem, 3M ESPE, St. Paul, MN), strictly observing the manufacturers’ instructions. The hydraulic pressure was released by small buccolingual rocking movements, and a 4 Kg load was applied for a period of 10 minutes. All specimens were left inside an incubator unit (BF 115; Binder GmbH, Tuttlingen, Germany) for a period of 24 hours in 100% humidity at a temperature of 36oC before testing. All clinical steps of the study were performed by the same clinician, while the laboratory procedures were undertaken by the same experienced dental technician.

The cement was allowed to polymerize for 72 hours before the specimens were cleared for any testing procedure. Room temperature (21 ± 2oC) and relative humidity (50 ± 10%) were monitored throughout the study.

**Figure 2:**
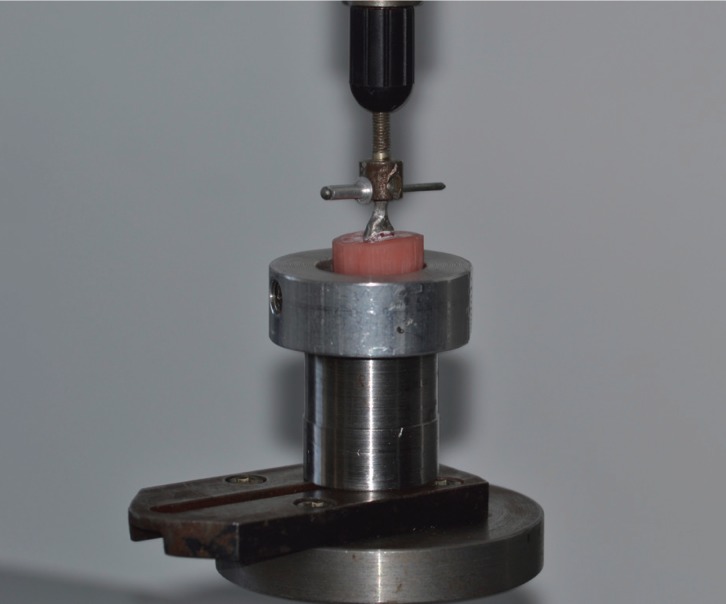
Specimen mounted on testing assembly for tensile load test.

Following the aforementioned period, each acrylic block containing the specimens was fixed inside a cylindrical custom-made aluminum mold, which in turn was mounted in a universal testing machine (AX M350-10KN; Testometric Co Ltd, Rochdale, UK). All specimens had a stainless-steel rod attached to the loop of the metal housings (Fig. 2). The testing machine exerted a gradually increasing force parallel to the long axes of the resin teeth, until failure occurred. For the tensile stress test a load cell of 500N was used with a crosshead speed of 1.0 mm/min.(30) Failure was defined as the point at which the tensile force reached a maximum value with subsequent debonding of the metal coping.

Descriptive statistics, one-way analysis of variance (ANOVA) (a=.05), and Tukey Honestly Significant Difference (HSD) (a=.05) were used to determine the effect of tensile failure loads among the tested groups of the study. Data were analyzed with the SPSS v21 software (SPSS, IBM Corp., Chicago, IL).

## Results

**Figure 3:**
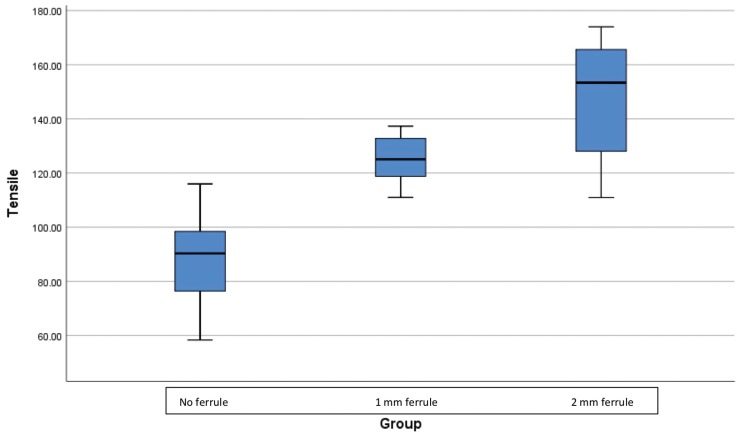
Boxplots of tensile failure loads (Newtons).

The results of the descriptive statistics for the tensile failure loads values of the different metal coping designs included in the study are depicted in  Table 1 and Figure 3. Mean tensile failure loads for the control group were 87.21 ± 18.26 N, while the 1 mm ferrule group recorded a higher mean failure load of 125.43 ± 8.79 N and the 2 mm ferrule group recorded the highest mean failure load of 146.12 ± 23.38 N. One-way ANOVA revealed significant differences (F= 28.04, *p*<0.001) in tensile failure loads between the groups tested ( Table 2). The Tukey HSD test revealed that all groups were significantly different (*p*<0.001-*p*=0.039), when compared in relation to their ferrule design ( Table 3). In all tested specimens, the luting agent failed. No debondings of the resin tooth analogs from the acrylic resin blocks were noted.

**Table 1:**
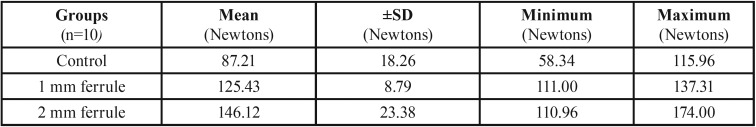
Descriptive statistics for tensile loads.

**Table 2:**
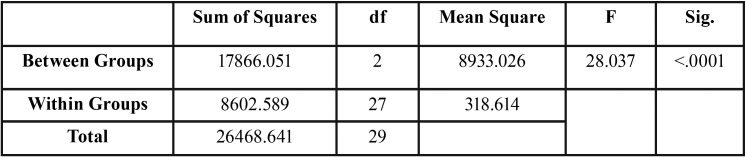
One-way ANOVA for tensile failure loads with different ferrule designs.

**Table 3:**
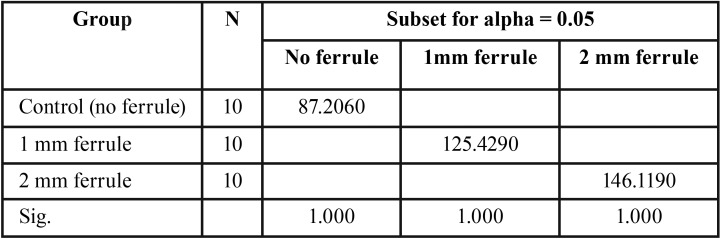
Tukey HSD test for tensile failure loads.

## Discussion

The results of the present study suggest that the null hypothesis should be rejected, as statistically significant differences could be detected in retention loads between the copings with and without a ferrule design. The retentive values for the 2 mm ferrule height design were significantly higher (*p*<0.001) than those of the no ferrule design (146.12 N vs 87.21 N). This finding could suggest that the beneficial effect of the ferrule might apply to overdenture abutment teeth in the same principle as in any other endodontically treated teeth with post and core restorations (27,28). The metal copings in the present study incorporated a metal lop for the attachment of the holding rod for the testing apparatus during the tensile stress measurements. This was implemented to simulate the tensile effect of a precision attachment during the intraoral removal phase. Although a plethora of overdenture attachments with various degrees of retention could be sourced through dental companies, the values obtained from the present study superseded all, thus strengthening the clinical relevance of the study design. Unfortunately, since this study’s experimental design was not utilized in any previous *in vitro* studies, no direct comparisons could be drawn. Nevertheless, these results should be verified with further *in vitro* investigations and with clinical studies.

The sole tooth utilized in the study was a mandibular canine, mainly in order to minimize any confounding variables and secondly because it has the highest survival rate in most of the clinical studies (11,13,20). Clinicians usually select mandibular premolars or canines as overdenture abutment teeth (11), which unlike implants, could be heterogenous regarding their endodontic anatomy, structure or periodontal condition. Specifically, mandibular canines have been, in most patients, strategically located at the corner of the arches, with long roots and generally single canals, which made them ideal abutment teeth for overdentures. The use of autopolymerizing PMMA resin to fabricate the resin teeth analogs has been advocated in a previous study design (30) as resin materials resemble the modulus of elasticity of the dentin. An additional advantage of the method employed was that all specimens had exactly the same dimensions.

Adhesive failure was recorded in all specimens of the present study. This finding was in agreement with the in vivo study by Chhabra *et al.*, (11) who reported that the loss of the metal coping ranked the highest of the coping related complications with 34% of the total overdenture abutments, although a coping design without an intraradicular dowel was utilized. A glass ionomer was used in the present study for the cementation of the metal copings. In the in vivo study by Gomez-Polo *et al.* (29) the most frequent complication (20%) was the dislodgement of the post. In their study the majority of the post and cores were also cemented with glass ionomer cements and restored with complete coverage fixed prostheses. The use of adhesive cementation protocols and resin cements would probably affect the final outcome.

A further limitation of this *in vitro* study was that all specimens were subjected to a static tensile load, which might not accurately represent intraoral conditions (30).The maximum load values which were recorded may have been smaller if a cyclic loading had been used. The gradual accumulation of small amounts of plastic strain produced by each testing cycle could lead to dissimilar failure loads than those recorded. However, even cyclic loading cannot represent the oral environment as a standardized load is preset throughout the testing procedure. Since mastication could be considered as a rather complex procedure influenced by many parameters, such as gender, age and occlusal scheme, the range of eccentric movements, opening and closing velocities, and directional changes could be extremely challenging to duplicate in an *in vitro* experimental procedure. Furthermore, the stresses induced by the overdenture prostheses on to the abutment teeth, the temperature changes in the oral environment, and chemomechanical and microbiologic influences might negatively affect the final outcome.

Future *in vitro* studies should test the influence of thermal cycling and fatigue loading on the retention of different metal overdenture coping designs. The influence of the overdenture base material should also be evaluated. The results of the present *in vitro* study can only offer an indication as to the retention failure loads of the specific designs and should be confirmed by well-designed, long-term prospective clinical trials.

## Conclusions

Within the limitations of the present *in vitro* study, the following conclusions can be drawn.

1. A metal coping provides better retention when a ferrule design preparation is applied, compared to a none ferrule preparation.

2. The height (2 mm vs 1 mm) of the ferrule design can significantly affect the retention of the metal copings.
